# Impact of acceleration treatment on treatment plan and delivery qualities in tomotherapy for lung cancer

**DOI:** 10.1002/acm2.70049

**Published:** 2025-02-20

**Authors:** Ryosuke Shirata, Tatsuya Inoue, Yugo Ebinuma, Akihiro Yamano, Takayuki Yagihashi, Hironori Nagata, Yumiko Minagawa, Yuki Mukai, Akiko Sato, Motoko Omura

**Affiliations:** ^1^ Department of Medical Physics Shonan Kamakura General Hospital Kamakura City Kanagawa Japan; ^2^ Department of Radiation Oncology Faculty of Medicine Juntendo University Bunkyo‐ku Tokyo Japan; ^3^ Juntendo University Graduate School of Medicine Bunkyo‐ku Tokyo Japan; ^4^ Department of Radiation Oncology Shonan Kamakura General Hospital Kamakura City Kanagawa Japan

**Keywords:** acceleration treatment, lung cancer, Precision TPS, tomotherapy, voxel‐less optimization ultra

## Abstract

**Background:**

Acceleration treatment (AT) is a novel treatment planning parameter introduced in the tomotherapy‐dedicated treatment planning system, Precision. This study explores the effects of AT on tomotherapy plans using helical (TomoHelical) and direct (TomoDirect) irradiation techniques.

**Methods:**

This study enrolled 20 patients with lung cancer. Initially, 10 TomoHelical and 10 TomoDirect treatment plans were created for each patient, utilizing patient‐specific field width and pitch with an AT setting of 0. These original plans were subsequently reoptimized by changing only the AT values to 1, 4, 7, and 10 without changing other calculation parameters to assess the impact of AT on dosimetric and delivery parameters. Additionally, the deliverability of all plans was evaluated through patient‐specific quality assurance using gamma analysis.

**Results:**

Increasing the AT from 0 to 10 led to a slight increase in maximum doses and a decrease in minimum doses within the target volume, thereby impairing dose homogeneity. Dose conformity to the target also deteriorated. Conversely, target coverage and delivery time improved considerably with higher AT values. Moreover, doses to organs at risk, including the lung, spinal cord, heart, and esophagus, remained clinically acceptable across all plans. Changes in these doses and the gamma pass rate in patient‐specific quality assurance were negligible with variations in AT. This trend was consistent across both delivery techniques.

**Conclusion:**

AT is a crucial parameter in tomotherapy planning for modulating plan and delivery qualities. Higher AT values can enhance target coverage and delivery time efficiency.

## 1 INTRODUCTION

Tomotherapy (Accuray Inc., Sunnyvale, CA, USA) is a 6‐MV flattening filter‐free linear accelerator that employs special dose delivery techniques for intensity‐modulated radiotherapy.^1^, ^2^ The radiation beam is dynamically shaped in the cranial‐caudal direction by moving jaws and in the lateral and anterior–posterior directions by a 64‐leaf binary multileaf collimator (MLC). Tomotherapy offers two modes of dose delivery: TomoHelical, which involves 360° gantry rotation, and TomoDirect, which delivers radiation at fixed, pre‐established angles.^3^, ^4^ During both radiation dose delivery modes, patients are moved through the gantry bore on a treatment couch. This unique delivery system enables highly conformal dose distributions to the target while minimizing exposure to clinical organs at risk (OARs). Numerous studies have demonstrated the effectiveness of both tomotherapy modes in treating a wide range of cancer types,^4^, ^5^, ^6^, ^7^, ^8^, ^9^, ^10^, ^11^, ^12^ including lung cancer.^13^, ^14^, ^15^, ^16^, ^17^, ^18^

Since its introduction to clinical practice, the tomotherapy machine has undergone several updates and modifications to enhance clinical outcomes and patient treatment efficiency.^4^ The latest model, Radixact, has increased its output to 1,000 monitor units per minute (MU/min), up from the 850 MU/min of earlier versions, leading to a considerable reduction in irradiation time. It has been reported that irradiation time with Radixact is approximately 30% shorter than with its predecessors.^4^ Parallel to the evolution of the tomotherapy machine, the dedicated treatment planning system (TPS) has also been updated. The previous TPS, known as Planning Station (Accuray Inc., Sunnyvale, CA, USA), incorporated the voxel‐less optimization (VoLO) system, which optimizes the dose using the beamlet coordinate system that passes through open MLC leaves, thereby reducing dose calculation time.^19^, ^20^ The latest TPS, Precision (Accuray Inc., Sunnyvale, CA, USA), introduced for Radixact, incorporates the VoLO‐ultra system. This system further decreases calculation time by performing dose calculations with a fluence convolved broad beam algorithm at each optimization iteration and using a collapsed cone convolution (CCC)‐superposition algorithm once every 20 iterations. Additionally, it employs the limited‐memory Broyden‐Fletcher‐Goldfarb‐Shanno Bound method for optimization, ensuring faster convergence to the global optimal solution.^21^ The use of Radixact with the VoLO‐ultra system has been reported to reduce calculation time by approximately 70% compared to the conventional VoLO system, according to Accuray's documentation.

During dose optimization, the patterns of the multileaf collimator (MLC) openings (open/close times) are adjusted to meet predefined dose objectives for the target and OARs. When the opening times of the MLC exhibit considerable variation, indicating substantial beam modulation complexity, the resulting MLC pattern tends to be intricate.^22^, ^23^ This complexity can lead to prolonged treatment times, causing discomfort for the patient and increasing the workload for medical staff. To address this issue, Boyd et al. proposed a method to reduce treatment time while preserving optimal dose distribution.^24^ This approach involves approximating the leaf open time (LOT) histogram of the treatment plan as a normal Gaussian distribution curve, then excluding MLC leaves with open times that deviate significantly from this curve during the optimization process. This methodology has been incorporated into the Precision TPS for Radixact as a parameter known as acceleration treatment (AT). In clinical practice, the selection of planning parameters, including field width (FW), pitch, and modulation factor (MF), is tailored to each patient and treatment site, often balancing the trade‐off between achieving superior dose distribution with longer treatment times and accepting less optimal dose distributions for shorter treatment durations. Although the MF is closely related to the quality of the dose distribution and treatment duration,^4^, ^25^ it is not available in the VoLO‐ultra system. Therefore, understanding the characteristics of the newly introduced AT parameter is crucial for clinical decision‐making and the development of effective treatment plans. To our knowledge, no study has yet explored the impact of AT in tomotherapy treatment planning for lung cancer. This study aims to examine the effects of AT in helical and direct tomotherapy plans, focusing on target dose coverage, conformity, homogeneity, OAR doses, and changes in delivery parameters, including treatment time and deliverability.

## 2 METHODS

### 2.1 Patient data

Twenty patients with lung cancer treated using tomotherapy at our institution between April 2021 and July 2022 were enrolled in this study. [Correction added on March 28 2025, after first online publication: The first sentence of section 2.1 has been updated.] The initial treatment plans were created using the previous Precision TPS (version 3.1.0.0) without the AT parameter for TomoHelical (10 patients) and TomoDirect (10 patients) modes. Lung cancer was selected as the treatment site because of the frequent application of both modes in treatment planning.^13^, ^14^, ^15^, ^16^, ^17^, ^18^ All computed tomography (CT) images were acquired under free‐breathing conditions using a Siemens 20‐slice CT scanner (Somatom Confidence, Siemens, Germany) with a reconstruction resolution of 0.98 × 0.98 × 2 mm^3^. The images were then transferred to RayStation TPS version 10A (Raysearch Laboratories, AB, Stockholm), where radiation oncologists delineated the gross tumor volume (GTV) and relevant OARs, including the total lungs, spinal cord, heart, and esophagus. The clinical target volume (CTV) was delineated by expanding the GTV by a 5 mm margin in all directions, excluding non‐invasive and bone regions. The planning target volume (PTV) was subsequently defined by expanding the CTV isotropically by a 5 mm margin. The CT image data and contoured structures were then transferred to the latest version of Precision TPS for treatment planning. Patient characteristics such as TNM stage, tumor location, tumor volume, and tumor dimensions in the cranial‐caudal direction, are summarized in Table 1.

**TABLE 1:** Patient characteristics and treatment planning parameters.

							Planning parameter		
	Patient	cTNM	Primary tumor location	Prescription (Gy/fraction)	Target volume (cc)	Target length in cranial‐caudal direction (mm)	Field width (cm)	Pitch	Beam angle (degree)
TomoHelical	H01	T4N1M0	Mediastinum	60/30 (40/20)	144.8	68.9	2.5	0.430	
	H02	T4N0M0	Left upper lobe	60/20	414.1	110.3	2.5	0.430	
	H03	T3N0M0	Left lower lobe	60/30	100.9	59.2	2.5	0.430	
	H04	T2bN0M0	Left lower lobe	60/20	231.9	83.6	2.5	0.430	
	H05	T2aN１M0	Mediastinum	66/33	60.2	96.4	2.5	0.430	
	H06	T1cN2M0	Mediastinum	66/33	103.3	79.3	2.5	0.430	
	H07	T1bN0M0	Right upper lobe	66/33	28.7	41.7	1.0	0.430	
	H08	T3N1M0	Left lower lobe	60/30	222.1	73.8	2.5	0.430	
	H09	T4N2M0	Right lower lobe	66/33 (44/22)	261.1	111.9	2.5	0.430	
	H10	T3N0M0	Left lower lobe	60/30	52.6	57.8	2.5	0.430	
TomoDirect	D01	T3N0M0	Left lower lobe	60/20	414.1	110.3	2.5	0.251	120, 140, 160, 180, 300, 320, 340
	D02	T1cN0M0	Right middle lobe	60/20	11.9	32.3	2.5	0.251	5, 20, 170, 185, 200, 215, 350
	D03	T1cN0M1a	Left upper lobe	60/30	193.3	109.3	5.0	0.500	5, 25, 145, 165, 185, 205, 345
	D04	‐	Left upper lobe	60/30	35.0	54.0	2.5	0.251	5, 10, 25, 30, 145, 165, 180, 185, 200, 205, 345, 350
	D05	T2N2M0	Right lower lobe	60/30	108.0	61.7	2.5	0.251	10, 30, 50, 160, 185, 210, 235, 350
	D06	T2aN0M0	Left lower lobe	66/33	60.9	52.1	2.5	0.251	20, 50, 150, 160, 185, 210, 235, 350
	D07	‐	Right upper lobe	60/20	13.8	30.7	2.5	0.251	15, 150, 180, 300, 325, 350
	D08	T1cN0M0	Right lower lobe	66/33	98.2	66.2	2.5	0.251	10, 30, 165, 185, 205, 225, 350
	D09	T4N2M0	Right upper lobe	60/30	455.3	112.6	2.5	0.251	0, 130, 160, 190, 300, 320, 340
	D10	T4N0M0	Right lower lobe	60/20	560.9	109.2	2.5	0.251	15, 145, 170, 195, 220, 300, 325, 350

### 2.2 Treatment planning

Treatment plans for either TomoHelical or TomoDirect mode were recreated for all patients using the latest version of Precision TPS (version 3.3.1.3). The TomoHelical plans were optimized using an FW of 1.0 cm (fixed‐jaw mode) and a pitch of 0.43 for patient H07 and an FW of 2.5 cm (dynamic‐jaw mode) and a pitch of 0.43 for the other nine patients. Furthermore, the TomoDirect plans were optimized using an FW of 5.0 cm (dynamic‐jaw mode) and a pitch of 0.50 for patient D03 and an FW of 2.5 cm (dynamic‐jaw mode) and a pitch of 0.251 for the other nine patients. An AT of 0 and a CCC algorithm were utilized for the dose optimization of all the plans. Optimization was conducted across three rounds, corresponding to 60 iterations, with the final calculation of the CCC algorithm performed at the end. Dose resolution was set as medium and high for optimization and final calculation, respectively. Detailed planning parameters for each patient are listed in Table 1. Prescription doses ranged from 60–66 Gy in 30–33 fractions. For patients H01 and H09, the prescription doses were adjusted to 40 Gy/20 fractions and 44 Gy/22 fractions, respectively, to account for the need for replanning due to tumor shrinkage. Postoptimization, the doses were normalized to the PTV with 50% of the prescription dose. Our institutional clinical goals for the target and OARs are detailed in Table 2. Dose objective parameters and weights were adjusted to minimize OAR doses without compromising target criteria.

**TABLE 2:** Clinical goals for treatment planning.

		Clinical goal
Target	PTV	*D*_95%_ ≧ 95% of prescription dose
		*D*_50%_ = Prescription dose
		*D*_max_ <110% of prescription dose
		
OAR	Lungs	*D*_mean_ ≦ 20 Gy
		V_20 Gy_ < 30%
	Spinal cord	*D*_max_ < 45 Gy
	Heart	*V*_30 Gy_ < 45%
		*V*_50 Gy_ < 25%
		*D*_mean_ ≦ 20 Gy
	Esophagus	*D*_max_ < 105% of prescription dose
		*D*_mean_ ≦ 34 Gy

After their creation, the original plans were reoptimized using AT parameters set to 1, 4, 7, and 10 with the same calculation and dose objective parameters as the original plans. The AT parameter truncates the upper tail of the LOT histogram, approximated as a Gaussian distribution, according to the input value (range: 0–10). For instance, an AT of 5 or 10 indicates that the maximum LOT is limited to values covering 95% (100 minus 5) or 90% (100 minus 10) of the Gaussian distribution, respectively. Notably, an AT of 0 corresponds to 99.5% (100 minus 0.5). All planning and optimization parameters, except for AT, were maintained as in the original plans to isolate the effects of the AT parameter. This study included a total of 100 treatment plans (five per patient).

### 2.3 Patient‐specific quality assurance

Patient‐specific quality assurance (PSQA) was conducted for all 100 plans utilizing the Delta4 phantom (Sun Nuclear, Melbourne, FL) to ascertain plan deliverability.^26^ PSQA plans were created on the Precision TPS according to the manufacturer's guidelines, and absolute dose calibration for Delta 4 was performed prior to each measurement session. The gamma passing rate (GPR) analysis, comparing measured to calculated doses, was carried out using the criteria of 3% dose difference (DD), 2‐mm distance to agreement, and a 10% threshold with global normalization, as recommended by AAPM TG218.^27^

### 2.4 Plan and delivery quality evaluations

To evaluate plan quality, various dosimetric parameters were calculated for the PTV, including the target coverage with 100% of the prescription dose (V_100%_), the near‐maximum dose (D_2%_), and the near‐minimum dose (D_98%_). Additionally, Paddick's conformity number (CN)^28^ and the homogeneity index (HI)^29^ were determined. The CN was calculated using the following equation:

(1) $$\mathrm{CN}\;=\frac{{\mathrm{PTV}}_{\mathrm{prescription}}^{2}}{\left(\mathrm{PTV}\;\times \;V_{\mathrm{prescription}}\right)}\;,$$

where PTV is the PTV volume, PTV_prescription_ is the PTV volume covered by the prescription dose, and V_prescription_ is the total volume receiving the prescription dose. The HI was calculated as follows:

(2) $$\mathrm{HI}\;=\frac{D_{2\%}-D_{98\%}}{D_{50\%}}\;,$$

where *D*_50%_ is the dose covering 50% of the PTV. Ideal values for CN and HI are close to 1 and 0, respectively. For OARs, the maximum dose (defined as D_2%_ for the spinal cord), the mean dose for the lungs, heart, and esophagus, and *V*_5Gy_ and *V*_20Gy_ for the lungs were computed. Delivery parameters, including beam on time, MF, gantry period, and maximum LOT for each AT plan, were also recorded. Furthermore, to investigate changes in plan and delivery qualities with variations in the AT parameter, relative or absolute differences in the evaluation indices were calculated using the plan with an AT of 0 as the reference.

### 2.5 Statistical analyses

All statistical analyses were performed using MATLAB 2020b (MathWorks Inc., Natick, MA, USA). Statistically significant differences in the evaluation parameters between each AT plan were calculated using one‐way ANOVA with the Bonferroni post hoc test. In all analyses, a *p*‐value of <0.05 indicated a statistically significant difference.

## 3 RESULTS

**FIGURE 1:**
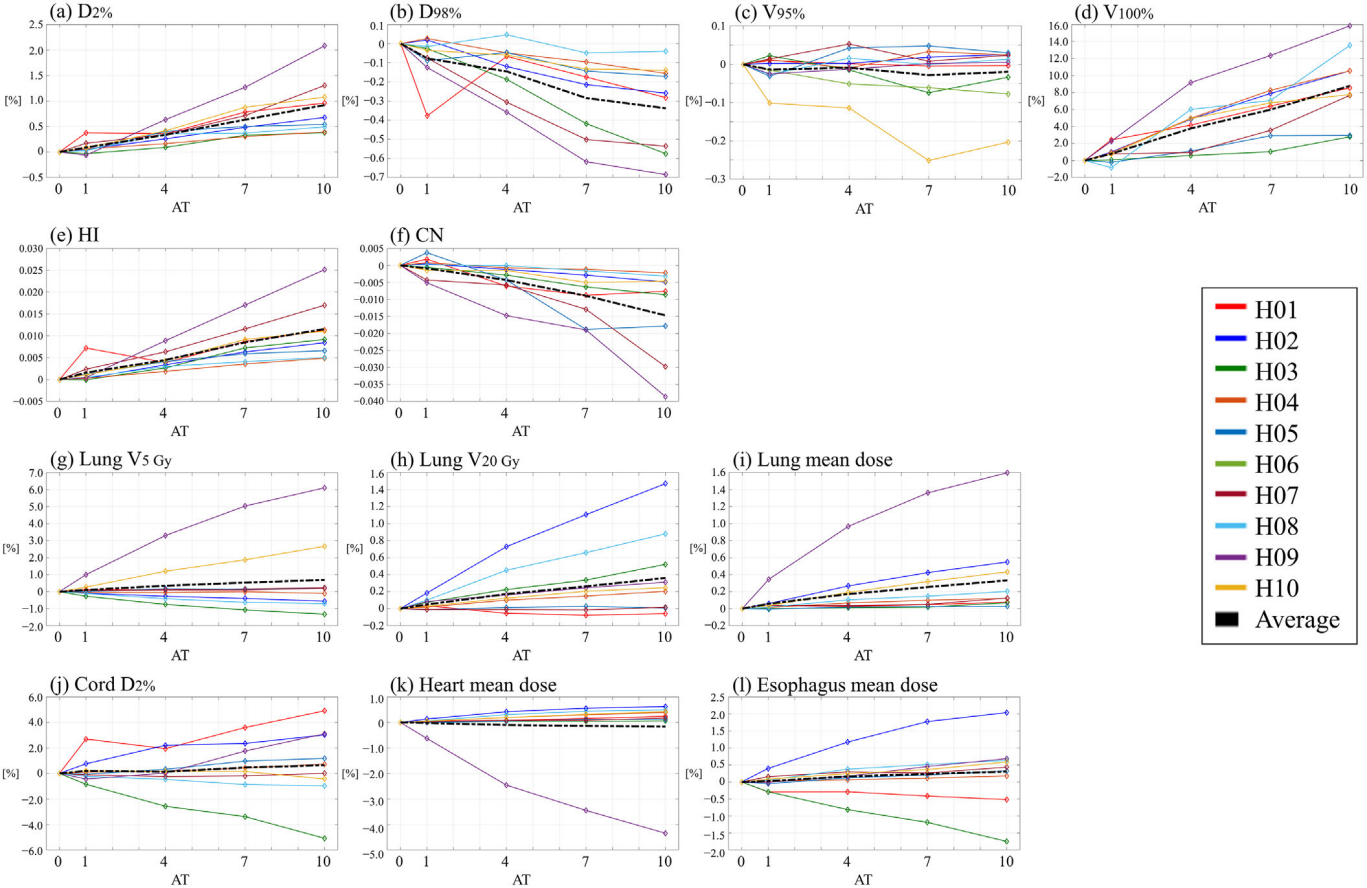
Dosimetric parameters of TomoHelical plans with AT values of 1, 4, 7, and 10, normalized to plans with AT 0 for 10 patients. Black dashed lines indicate the average dosimetric parameters across the patients. Panels: (a) near‐maximum dose for PTV (*D*_2%_), (b) near‐minimum dose for PTV (*D*_98%_), (c) target coverage for PTV (*V*_95%_), (d) target coverage for PTV (*V*_100%_), (e) homogeneity index for PTV (HI), (f) conformity number for PTV (CN), (g) lung *V*_5Gy_, (h) lung *V*_20 Gy_, (i) mean lung dose, (j) near‐maximum spinal cord dose (*D*_2%_), (k) mean heart dose, and (l) mean esophagus dose.

**FIGURE 2:**
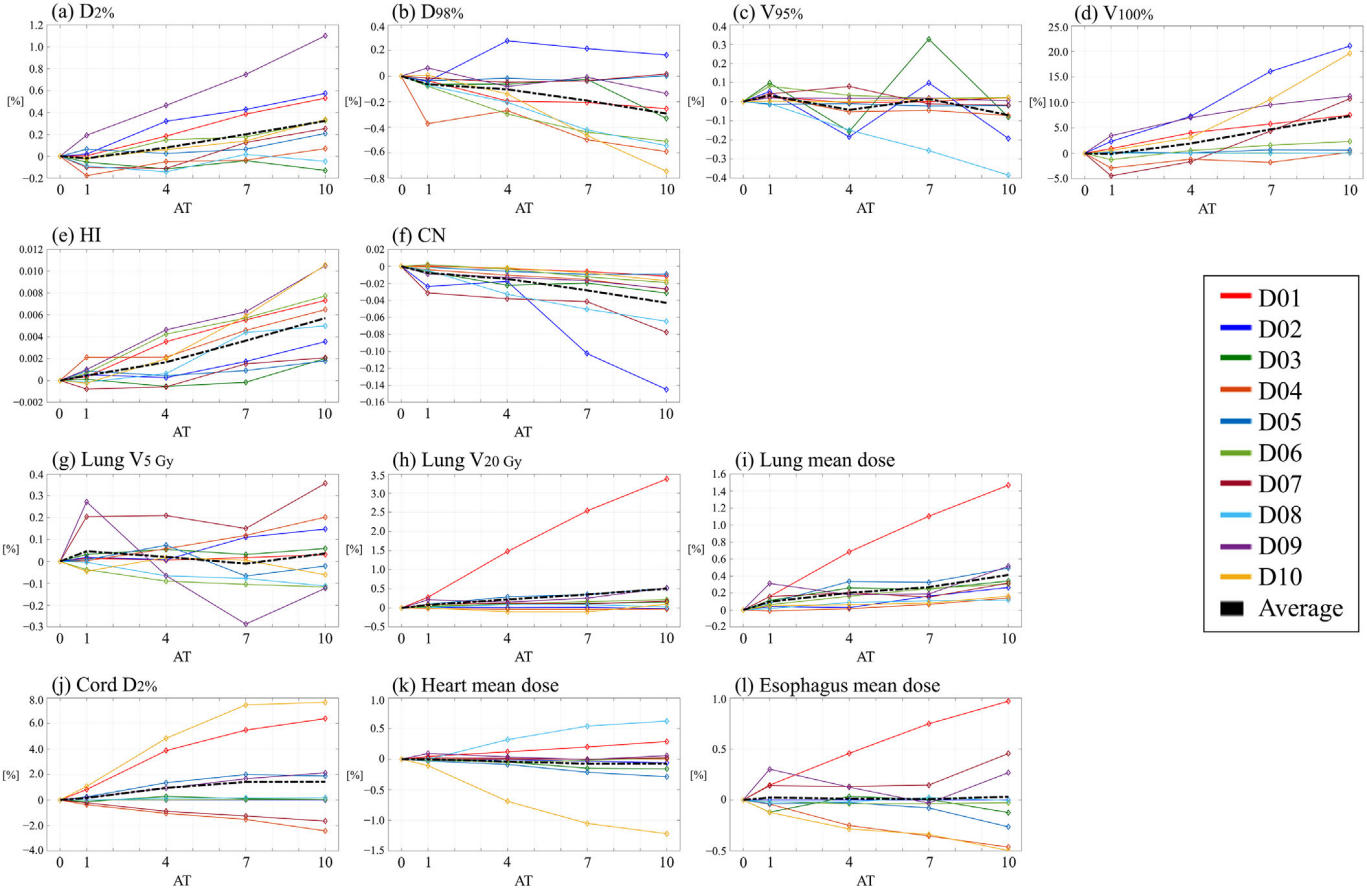
Dosimetric parameters of TomoDirect plans with AT values of 1, 4, 7, and 10, normalized to plans with AT 0 for 10 patients. Black dashed lines indicate the average dosimetric parameters across the patients. Panels include: (a) near‐maximum dose for PTV (D_2%_), (b) near‐minimum dose for PTV (D_98%_), (c) target coverage for PTV (V_95%_), (d) target coverage for PTV (V_100%_), (e) homogeneity index for PTV (HI), (f) conformity number for PTV (CN), (g) lung *V*_5 Gy_, (h) lung *V*_20 Gy_, (i) mean lung dose, (j) near‐maximum spinal cord dose (D_2%_), (k) mean heart dose, and (l) mean esophagus dose.

All plans with varying AT values met the clinical criteria for the target and OARs. Tables S1 and S2 in the supplementary material summarize the patient‐specific dosimetric parameters for TomoHelical and TomoDirect plans, respectively. Figures 1 and 2 illustrate the variation in dosimetric parameters as a function of the AT parameter for TomoHelical and TomoDirect plans, respectively. For the averaged variations, when D_2%_, *V*_100%_, HI, lung *V*_20Gy_, and lung mean dose were increased, *D*_98%_ and CN decreased; however, *V*_95%_, lung *V*_5Gy_, Cord *D*_2%_, heart mean dose, and esophagus mean dose did not change with increasing AT.

For the TomoHelical and TomoDirect plans, Table 3 provides the mean values and standard deviations of the differences in dosimetric parameters for each AT plan compared with the AT0 plan across 10 patients, while Table 4 summarizes the statistical values for the differences in the evaluated dosimetric parameters between each AT plan. The mean differences in D_2%_, D_98%_ for PTV, and OAR doses, excluding the spinal cord for TomoDirect plans, remained within 1% between AT0 and AT10 plans. However, significant differences were observed in D_2%_ between TomoHelical‐AT0 and ‐AT10 plans and D_2%_ and D_98%_ between TomoDirect‐AT0 and ‐AT10 plans. With respect to the OARs, a significant difference was observed for only the lung mean dose between TomoDirect‐AT0 and ‐AT10 plans. The average maximum dose for the spinal cord in TomoDirect‐AT7 and ‐AT10 plans increased by 1.4% compared to AT0 plans, although no significant difference was observed. The homogeneity index (HI) and Paddick's conformity number (CN) deteriorated with increasing AT values, with maximum average changes of 0.012 and −0.01 for TomoHelical plans and 0.006 and −0.04 for TomoDirect plans, respectively. Significant differences were observed in HI between TomoHelical‐AT0 and ‐AT10 plans and HI and CN between TomoDirect‐AT0 and ‐AT10 plans. Regarding target coverage (V_100%_), this metric significantly improved with increasing AT values. On average, V_100%_ for TomoHelical‐ and TomoDirect‐AT10 plans was 8.8% and 7.3% higher, respectively, than that for AT0 plans. Furthermore, no significant difference was observed for V_95%_. Tables S3 and S4 in the supplementary material present the differences in the dosimetric parameters for each AT plan compared to the AT0 plan for individual patients.

**TABLE 3:** Mean and standard deviation of dosimetric differences between TomoHelical/TomoDirect plans across 10 patients.

		PTV						Lung			Cord	Heart	Esophagus
		*D_2_*_%_(%)	*D*_98%_ (%)	*V*_95%_ (%)	*V*_100%_ (%)	HI	CN	*V*_5 Gy_ (%)	*V*_20 Gy_ (%)	Mean dose (%)	*D*_2%_ (%)	Mean dose (%)	Mean dose (%)
TomoHelical	AT0	–	–	–	–	–	–	–	–	–	–	–	–
	AT1	0.1 ± 0.1	−0.1 ± 0.1	0.0 ± 0.0	0.8 ± 1.0	0.002 ± 0.002	0.00 ± 0.00	0.1 ± 0.3	0.0 ± 0.1	0.1 ± 0.1	0.2 ± 1.0	0.0 ± 0.2	0.0 ± 0.2
	AT4	0.3 ± 0.1	−0.1 ± 0.1	0.0 ± 0.0	3.8 ± 2.8	0.004 ± 0.002	0.00 ± 0.00	0.3 ± 1.2	0.2 ± 0.2	0.2 ± 0.3	0.1 ± 1.3	−0.1 ± 0.8	0.2 ± 0.5
	AT7	0.6 ± 0.3	−0.3 ± 0.2	0.0 ± 0.1	6.0 ± 3.3	0.009 ± 0.004	−0.01 ± 0.01	0.5 ± 1.8	0.3 ± 0.4	0.3 ± 0.4	0.5 ± 1.9	−0.1 ± 1.2	0.2 ± 0.7
	AT10	0.9 ± 0.5	−0.3 ± 0.2	0.0 ± 0.1	8.8 ± 4.1	0.012 ± 0.006	−0.01 ± 0.01	0.7 ± 2.2	0.4 ± 0.5	0.3 ± 0.5	0.6 ± 2.7	−0.2 ± 1.5	0.3 ± 1.0
TomoDirect	AT0	–	–		–	–	–	–	–	–	–	–	–
	AT1	0.0 ± 0.1	−0.1 ± 0.1	0.0 ± 0.0	−0.1 ± 2.3	0.000 ± 0.001	−0.01 ± 0.01	0.0 ± 0.1	0.1 ± 0.1	0.0 ± 0.0	0.2 ± 0.5	0.0 ± 0.1	0.0 ± 0.1
	AT4	0.1 ± 0.2	−0.1 ± 0.2	0.0 ± 0.1	1.9 ± 3.2	0.002 ± 0.002	−0.01 ± 0.01	0.0 ± 0.1	0.2 ± 0.5	0.1 ± 0.1	0.9 ± 1.9	0.0 ± 0.3	0.0 ± 0.2
	AT7	0.2 ± 0.2	−0.2 ± 0.2	0.0 ± 0.1	4.7 ± 5.8	0.004 ± 0.002	−0.03 ± 0.03	0.0 ± 0.1	0.4 ± 0.8	0.1 ± 0.2	1.4 ± 2.9	−0.1 ± 0.4	0.0 ± 0.3
	AT10	0.3 ± 0.4	0.3 ± 0.3	−0.1 ± 0.1	7.3 ± 8.1	0.006 ± 0.003	−0.04 ± 0.04	0.0 ± 0.2	0.5 ± 1.0	0.2 ± 0.2	1.4 ± 3.3	−0.1 ± 0.5	0.0 ± 0.4

**TABLE 4:** Statistical values for differences in dosimetric and delivery parameters between each AT plan in TomoHelical/TomoDirect.

		*p*‐value										
TomoHelical		one‐way ANOVA	AT 0 vs. 1	AT 0 vs. 4	AT 0 vs. 7	AT 0 vs. 10	AT 1 vs. 4	AT 1 vs. 7	AT 1 vs. 10	AT 4 vs. 7	AT 4 vs. 10	AT 7 vs. 10
PTV	D_2%_	<0.001			<0.001	<0.001		0.001	<0.001		<0.001	
	D_98%_	<0.001			0.002			0.006				
	V_95%_											
	V_100%_	<0.001		0.032	<0.001	<0.001		<0.001	<0.001		<0.001	
	HI	<0.001			<0.001	<0.001		0.001	<0.001		<0.001	
	CN	<0.001			<0.001			<0.001			0.02	
Lung	V_5Gy_											
	V_20Gy_											
	Mean dose											
Cord	D_2%_											
Heart	Mean dose											
Esophagus	Mean dose											
	Pass rate											
	MF	<0.001	0.027	<0.001	<0.001	<0.001	<0.001	<0.001	<0.001	<0.001	<0.001	
	Beam on time	<0.001	<0.001	<0.001	<0.001	<0.001	<0.001	<0.001	<0.001	<0.001	<0.001	0.005
	Gantry period	<0.001		<0.001	<0.001	<0.001	0.015	<0.001	<0.001			
	Max LOT	<0.001	<0.001	<0.001	<0.001	<0.001	<0.001	<0.001	<0.001	<0.001	<0.001	0.008

		*p*‐value										
TomoDirect		one‐way ANOVA	AT 0 vs. 1	AT 0 vs. 4	AT 0 vs. 7	AT 0 vs. 10	AT 1 vs. 4	AT 1 vs. 7	AT 1 vs. 10	AT 4 vs. 7	AT 4 vs. 10	AT 7 vs. 10
PTV	D_2%_	0.008				0.033			0.032			
	D_98%_	0.021				0.022						
	V_95%_											
	V_100%_	0.007				0.029			0.032			
	HI	<0.001			0.002	<0.001		0.028	<0.001		<0.001	
	CN	0.005				0.011						
Lung	V_5Gy_											
	V_20Gy_											
	Mean dose	0.008				0.006						
Cord	D_2%_											
Heart	Mean dose											
Esophagus	Mean dose											
	Pass rate											
	MF	<0.001		<0.001	<0.001	<0.001		<0.001	<0.001		0.005	
	Beam on time	<0.001		<0.001	<0.001	<0.001	0.004	<0.001	<0.001		<0.001	
	Max LOT	<0.001		<0.001	<0.001	<0.001	0.004	<0.001	<0.001		<0.001	

**FIGURE 3:**
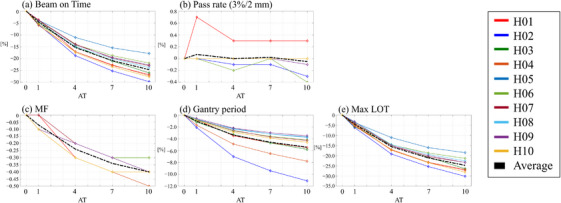
Delivery parameters and gamma pass rate for TomoHelical plans with AT values of 1, 4, 7, and 10, normalized to plans with AT 0 for 10 patients. Black dashed lines indicate the averaged delivery parameters across the patients. Parameters include: (a) beam on time (Time), (b) gamma pass rate (Pass rate), (c) actual modulation factor (MF), (d) gantry period, and (e) maximum leaf open time (max LOT).

**FIGURE 4:**
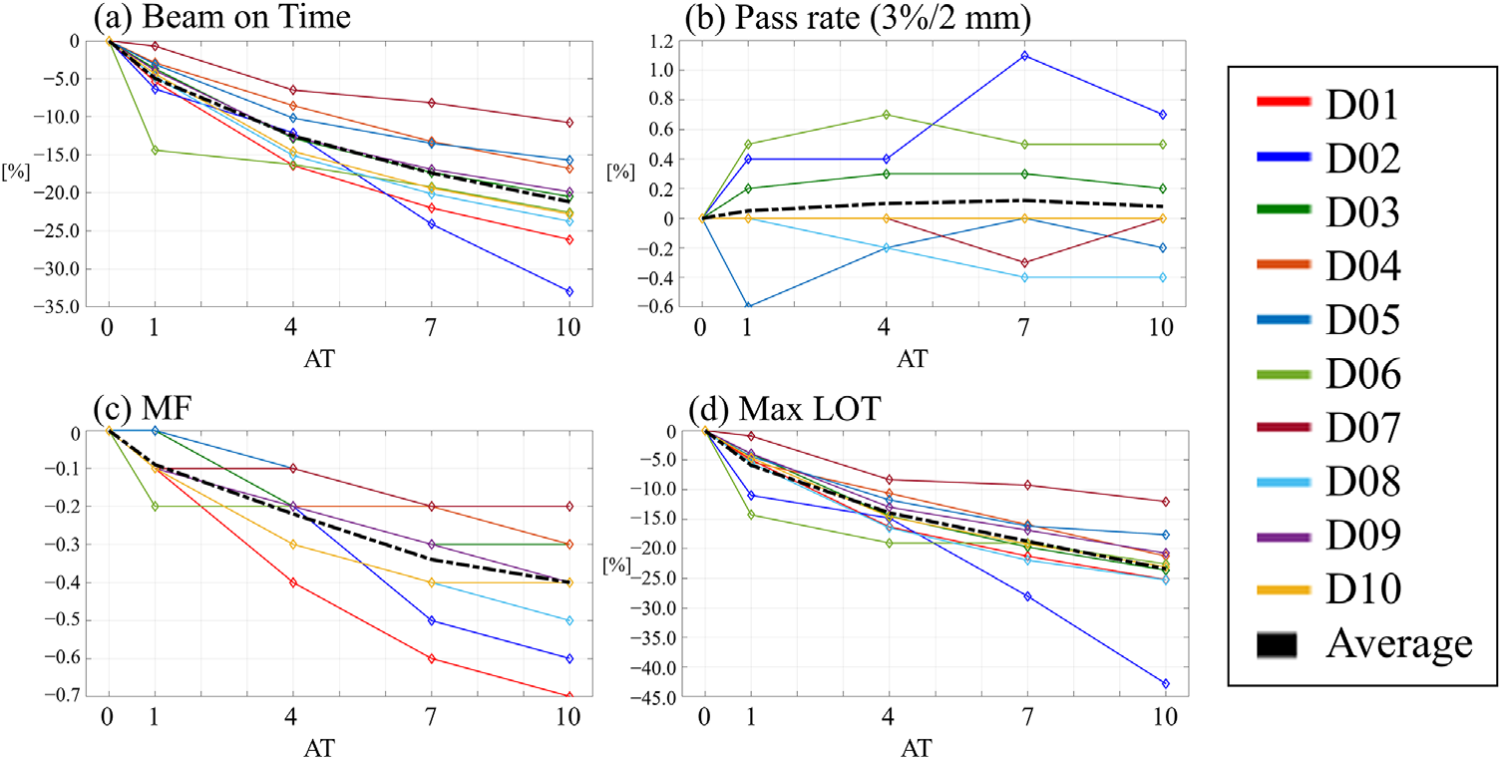
Delivery parameters and gamma pass rate for TomoDirect plans with AT values of 1, 4, 7, and 10, normalized to plans with AT 0 for 10 patients. Black dashed lines indicate the averaged delivery parameters across the patients. Parameters include: (a) beam on time (Time), (b) gamma pass rate (Pass rate), (c) actual modulation factor (MF), and (d) maximum leaf open time (max LOT).

Tables S5 and S6 presented in the supplementary material detail the patient‐specific GPR, MF, beam on time, and maximum LOT for TomoHelical and TomoDirect plans, respectively, along with the gantry period for TomoHelical plans. Figures 3 and 4 depict the variation in delivery parameters and GPR as a function of the AT parameter for TomoHelical and TomoDirect plans, respectively. For the averaged variations, beam on time, modulation factor, gantry period, and maximum leaf open time decreased; however, no change was observed in the gamma pass rate with increasing AT.

Table 5 lists the mean values and standard deviations of the differences in GPR and delivery parameters for each AT plan compared to the AT0 plan across 10 patients in TomoHelical and TomoDirect plans. Table 4 summarizes the statistical values for the differences in evaluated delivery parameters between each AT plan in TomoHelical and TomoDirect. As the AT parameter increased, a significant decrease was observed in MF, beam on time, gantry period, and maximum LOT. The average reduction rates in beam on time between AT0 and AT10 plans were 24.7% and 21.2% for TomoHelical and TomoDirect plans, respectively. Meanwhile, the deviation in GPR for the 3%/2 mm criteria was insignificant, with an average change of 0.1% between AT0 and AT10 plans. In TomoHelical plans, the gantry speed per rotation was on average 1.0–5.4 s faster with increasing AT compared to the AT0 plan. Tables S7 and S8 presented in the supplementary material show the differences in GPR and delivery parameters for each AT plan compared to the AT0 plan.

**TABLE 5:** Mean and standard deviation of delivery differences and GPR for TomoHelical/TomoDirect across 10 patients.

		Pass rate 3%/2 mm(%)	Modulation factor	Beam on time (%)	Gantry period (s)	Max LOT (%)
TomoHelical	AT0	–	–	–	–	–
	AT1	0.1 ± 0.2	−0.1 ± 0.0	−4.6 ± 0.9	−1.0 ± 0.5	−4.5 ± 1.0
	AT4	0.0 ± 0.1	−0.2 ± 0.1	−15.4 ± 2.2	−3.4 ± 1.5	−15.6 ± 2.2
	AT7	0.0 ± 0.1	−0.3 ± 0.1	−20.9 ± 2.8	−4.6 ± 2.0	−21.1 ± 2.8
	AT10	0.0 ± 0.2	−0.4 ± 0.1	−24.7 ± 3.5	−5.4 ± 2.4	−24.8 ± 3.5
TomoDirect	AT0	–	–	–	–	–
	AT1	0.1 ± 0.3	−0.1 ± 0.1	−4.9 ± 3.7	–	−5.9 ± 3.9
	AT4	0.1 ± 0.3	−0.2 ± 0.1	−12.5 ± 3.3	–	−14.0 ± 3.1
	AT7	0.1 ± 0.4	−0.3 ± 0.1	−17.4 ± 4.7	–	−18.8 ± 4.9
	AT10	0.1 ± 0.3	−0.4 ± 0.2	−21.2 ± 6.1	–	−23.4 ± 7.9

## 4 DISCUSSION

This study explored the effects of the newly introduced treatment planning parameter, AT, on plan and delivery qualities in lung cancer treatment utilizing TomoHelical and TomoDirect techniques. In traditional tomotherapy treatment planning, plan quality is predominantly regulated by MF and pitch, defined as the maximum leaf opening time divided by the average (nonzero) leaf opening time and the travel distance of the treatment couch for a complete gantry rotation with respect to the beam width at the axis of rotation, respectively. These factors are directly associated with the intensity modulation of the radiation beam.^4^ Numerous studies have confirmed that an increase in MF and pitch leads to enhanced dose distribution across various treatment sites.^25^, ^30^, ^31^, ^32^, ^33^, ^34^, ^35^ However, this improvement in plan quality comes at the cost of increased delivery time; a higher MF and a smaller pitch, which introduces a large overlap region between adjacent fan beams, resulting in longer delivery times. Extended delivery durations can exacerbate the negative impact of patient body movement and breathing motion, potentially resulting in dose discrepancies or the interplay effect between target motion and beam intensity modulation.^36^, ^37^ Additionally, baseline drift in breathing motion is often observed in prolonged treatments.^38^ Due to its unique slice‐by‐slice delivery method coupled with a moving couch, tomotherapy is particularly vulnerable to such motions.^39^ Consequently, managing delivery time while preserving high plan quality is essential for successful tomotherapy treatment.

RayStation TPS (version 10A) for tomotherapy recently incorporated an optimization technique based on delivery time constraints. Yagihashi et al. reported on the impact of a new planning parameter, the delivery time factor, on plan quality and delivery time.^40^ Similarly, the latest Precision TPS (version 3.3.1.3) introduced the AT parameter, which is associated with delivery time constraints instead of the MF.^24^ Our study revealed that adjusting the AT from 0 to 10 resulted in significantly enhanced treatment time efficiency, with a reduction in estimated delivery time ranging from 17.9% to 29.8% (24.7% on average) for TomoHelical plans and 10.8% to 33.0% (21.2% on average) for TomoDirect plans. Furthermore, there was no considerable increase in the doses to OARs from the AT0 plans, except for the maximum dose to the spinal cord in TomoDirect plans. Specifically, the dose increased by 4.6 Gy for patient D10, which was still deemed clinically acceptable by radiation oncologists. Notably, the deviation in the OAR doses by change in the AT might change depending on the planner's intention. If planners prefer much lower OAR doses, stricter plans can be created by strengthing the dose objectives and/or objective weights for OARs. Along with this, the MF increases, resulting in the further deviation in the OAR doses. In practice, our previous study demonstrated that increasing MF could reduce the OAR doses in lung cancer tomotherapy planning.^40^ Intriguingly, while the maximum and minimum doses in the target volume slightly increased and decreased, respectively, leading to poorer dose homogeneity, the target coverage improved with increasing AT. This improvement in target coverage may be attributed to the weakening of dose constraints to OARs surrounding the target due to reduced intensity modulation with higher AT values. Moreover, increasing AT led to the removal of larger MLC components, potentially resulting in suboptimal dose distributions.^22^, ^23^, ^41^, ^42^ PSQA was conducted for all AT plans, and the GPR results were compared, revealing insignificant variation with changes in AT. Based on these findings, using a higher AT value may be warranted for treatment time efficiency in clinical practice if there is no need to compromise target coverage and sparing of OARs.

A noteworthy observation was a unique case where dosimetric and delivery parameters remained unchanged despite variations in AT due to the gantry rotation speed reaching its maximum (11.8 s per rotation). In such scenarios, changes in AT will not affect the maximum LOT, and planners should consider gantry speed and pitch when creating treatment plans with AT parameters.

One limitation of this study is the small patient cohort. Consequently, while a general trend in the variation of dosimetric and delivery parameters with changes in AT was identified, a patient‐wise correlation between AT and the magnitude of variations could not be established. Notably, although the characteristics of the changes in dosimetric and delivery parameters with changes in the AT were similar for the TomoHelical and TomoDirect modes, there was a distinct difference in the irradiation technique between the two. For TomoDirect mode, constructing the LOT histogram and removing MLC components by AT values were undertaken for every beam angle. Thus, the impact of dosimetric and delivery consequences may differ between patients depending on the angles utilized. Expanding the patient cohort could further elucidate the patient‐specific impact of AT based on factors such as irradiation mode, target volume and location, and the physical distance between the target and OARs, thereby reinforcing the findings of this study. Additionally, the effects of AT on treatment sites other than the lung were not examined in this study; however, these effects warrant investigation in future research.

## 5 CONCLUSIONS

In summary, this study demonstrated that AT is a pivotal planning parameter in tomotherapy, applicable to TomoHelical and TomoDirect modes for lung cancer treatment. Increasing the AT value was associated with enhanced target coverage and delivery efficiency, while simultaneously maintaining OAR sparing and dose deliverability.

## CONFLICT OF INTEREST STATEMENT

The authors declare no conflicts of interest.

## Supporting information



Supporting information
