# Supplementary material for: Impact of acceleration treatment on treatment plan and delivery qualities in tomotherapy for lung cancer
Source: J Appl Clin Med Phys. 2025 Feb 20;26(5):e70049. doi: 10.1002/acm2.70049 (PMC12059278; doi:10.1002/acm2.70049)
Supplement: Supplementary file 1 — Supporting information [file ACM2-26-e70049-s001.docx]

Table S1. Patient-specific dosimetric parameters for TomoHelical plans with AT = 0, 1, 4, 7, 10

Table S2. Patient-specific dosimetric parameters for TomoDirect plans with AT = 0, 1, 4, 7, 10

Table S3. Patient-specific dosimetric parameters for TomoHelical plans with AT = 1, 4, 7, 10, normalized to plan with AT = 0

Table S4. Patient-specific dosimetric parameters for TomoDirect plans with AT = 1, 4, 7, 10, normalized to plan with AT = 0

Table S5. Patient-specific delivery parameters and gamma pass rate for TomoHelical plans with AT = 0, 1, 4, 7, 10

Table S6. Patient-specific delivery parameters and gamma pass rate for TomoDirect plans with AT = 0, 1, 4, 7, 10

Table S7. Patient-specific delivery parameters and gamma pass rate for TomoHelical plans with AT = 1, 4, 7, 10, normalized by plan with AT = 0

Table S8. Patient-specific delivery parameters and gamma pass rate for TomoDirect plans with AT = 1, 4, 7, 10, normalized by plan with AT = 0
